# Rapid, inexpensive fabrication of electrophoretic microdevices for fluorescence detection

**DOI:** 10.1002/elps.202200090

**Published:** 2022-07-08

**Authors:** Daniel A. Nelson, Brandon L. Thompson, An‐Chi Scott, Renna Nouwairi, Christopher Birch, Jacquelyn A. DuVall, Delphine Le Roux, Jingyi Li, Brian E. Root, James P. Landers

**Affiliations:** ^1^ Department of Chemistry University of Virginia Charlottesville Virginia USA; ^2^ Department of Mechanical Engineering University of Virginia Charlottesville Virginia USA; ^3^ Department of Pathology University of Virginia Charlottesville Virginia USA

**Keywords:** deoxyribonucleic acid, DNA, electrophoresis

## Abstract

The laser print, cut, and laminate (PCL) method for microfluidic device fabrication can be leveraged for rapid and inexpensive prototyping of electrophoretic microchips useful for optimizing separation conditions. The rapid prototyping capability allows the evaluation of fluidic architecture, applied fields, reagent concentrations, and sieving matrix, all within the context of using fluorescence‐compatible substrates. Cyclic olefin copolymer and toner‐coated polyethylene terephthalate (tPeT) were utilized with the PCL technique and bonding methods optimized to improve device durability during electrophoresis. A series of separation channel designs and centrifugation conditions that provided successful loading of sieving polymer in less than 3 min was described. Separation of a 400‐base DNA sizing ladder provided calculated base resolution between 3 and 4 bases, a greater than 18‐fold improvement over separations on similar substrates. Finally, the accuracy and precision capabilities of these devices were demonstrated by separating and sizing DNA fragments of 147 and 167 bases as 148.62 ± 2 and 166.48 ± 3 bases, respectively.

**Abbreviations**
B: buffer
BW: buffer waste
COC: cyclic olefin copolymer
DIPS: dual‐integrated Peltier system
*L*_eff_
: effective separation length
LP: long pass
PC: polycarbonate
PCL: print, cut, and laminate
PeT: polyethylene terephthalate
PLA: poly(lactic acid)
*R*_bp_
: base‐pair resolution
*R*_s_
: electrophoretic resolution
S: sample
SW: sample waste
tPeT: toner‐coated polyethylene terephthalate

## 1 INTRODUCTION

Microchip electrophoresis is a powerful alternative to traditional capillary electrophoresis as it provides opportunities for reduced reagent cost, potential portability, and decreased analysis time [1]. To perform capillary sieving electrophoresis on microfluidic devices, the optimization of multiple parameters (e.g., fluidic architecture, sieving matrix composition and concentration, voltages, and sample‐to‐reagent ratios) is required. When optimizing these conditions on microdevices, rapid, inexpensive prototyping, and reusability are ideal.

Glass microdevices offer reusability but require clean room facilities for photolithography and chemical etching in a laborious multiple‐step process that is costly and, frankly, impractical for rapid prototyping [2]. Alternatively, microdevices can be made from cost‐effective polymeric materials, such as poly(methyl methacrylate), cyclic olefin copolymer (COC), and polycarbonate, which have desirable optical properties for fluorescence detection of analytes and are amenable to rapid prototyping techniques (e.g., milling, embossing, and injection molding) [3]. However, these materials typically are not as robust as glass from a “reusability” perspective, thus fabricating iteratively improved designs is essential when prototyping fluidic architectures for optimized electrophoresis.

A method for rapid, inexpensive microdevice fabrication was reported by do Lago et al., whereby printer toner ink was selectively deposited on thin sheets of polyethylene terephthalate (PeT) that were then bonded via lamination; the areas devoid of toner became the microfluidic channels that were adequate in dimension and physicochemical properties for electrophoresis [4]. This revolutionary report presented a fast and cost‐effective fabrication method for electrophoretic microfluidic devices, albeit, with channels limited in depth by the number of polymeric layers and the number of ∼6‐µm toner layers utilized. Research from our group expanded on this concept and created multilayer devices by laser ablating through toner‐coated PeT (tPeT) layers to create fluidic architecture limited only by the combined number of ablated PeT and tPeT layers that could be bonded through simple lamination [5, 6]. We have defined this fabrication approach as the laser print, cut, and laminate (PCL) method [7], which is ideal for rapid microfluidic prototyping owing to the simplicity of the processes, and low materials cost. Work implementing the PCL method created centrifugal microdevices for DNA extraction [6], purification [8], and multiplexed amplification [9] with the integration of numerous elements for fluidic control [10]. However, the PCL method has not been expanded to develop microdevices for electrophoresis and fluorescence detection.

Combining PCL and microchip electrophoresis presents new potential for quickly prototyping and optimizing electrophoresis parameters. The first DNA separations on PeT/tPeT devices demonstrated that the separation of DNA fragments with a resolution of 56 bases was possible using PCL‐compatible substrates [11]. This was a milestone achievement as an analytical electrophoretic platform, primarily because it demonstrated that these inexpensive substrates were biocompatible and did not exhibit insurmountable problems with fluorescence detection. However, this level of resolution would be limited to the analysis of PCR products differing greatly in molecular size and would have to be dramatically improved to increase applicability to a wider range of chemical and biological applications like gene expression analysis and genotyping.

Here, we demonstrate that the limitations reported in prior literature for DNA fragment analysis on PeT microdevices can, in fact, be overcome. We describe the results from examining the inherent fluorescence, or autofluorescence, of multiple polymeric materials and different bonding conditions to optimize the fabrication of microfluidic electrophoresis chips via the PCL method. Various architectural designs were evaluated with results demonstrating successful polymer loading by centrifugation in a reasonable amount of time. Additionally, the shape of the electrokinetically injected sample plug in the differing architectures was examined with respect to effects on resolution. Finally, we demonstrate a successful separation of a DNA sizing standard and forensically relevant short tandem repeat (STR) markers with an 18‐fold improvement in resolution over previously reported separations on PCL devices.

## 2 MATERIALS AND METHODS

### 2.1 Microfluidic chip fabrication

The three‐layer microfluidic devices were made via the print, cut, and laminate method, previously described by Thompson et al. [7], using a combination of COC (Zeon Chemicals, Louisville, KY, USA) and toner‐coated polyethylene terephthalate (tPeT). Sheets of PeT (Film Source Inc., MO, USA) were coated twice on each side with toner (HP C‐4127X) using a laser printer (HP Laserjet 400). A single layer of toner was 6‐µm deep, making each tPeT layer 124‐µm thick (four 6‐µm layers of toner in a 100‐µm PeT sheet). AutoCAD software was used to create several different iterations of the microfluidic device. A CO_2_ laser system (VLS3.50, Universal Laser Systems, AZ, USA) was used to ablate the layers according to the CAD design. As shown in Figure S1, the top COC layer, layer 1, consisted of alignment holes and vents that enable access to the fluidic channels in the subsequent layer. Layer 2, the middle layer of the device, was made from tPeT and contained microfluidic architecture for electrophoretic separation and holes for alignment. Finally, layer 3, the bottom layer, only consisted of alignment holes. The rectangular microfluidic chips were 80‐mm long and 40‐mm wide. Following laser ablation, the three layers were stacked in alignment for bonding and sent through a heated roll laminator at 200°C (Model 305, Mega Dry Film Laminator, Cambridge, UK). Bonding studies compared laminating the COC and tPeT layers with and without the plasma oxidization of individual layers prior to lamination. Following fabrication, small reservoirs for the sample (S), sample waste (SW), buffer (B), and buffer waste (BW) were 3D printed (Dremel Digilab 3D45, USA) from poly(lactic acid) (3DXTech, MI, USA) and attached to the devices using 5 minute epoxy (Devcon, MA, USA).

### 2.2 Instrumentation

#### 2.2.1 DIPS system

To centrifugally load reagents into the microdevice, a dual‐integrated Peltier spin (DIPS) system, described elsewhere [5], was used for centrifugal polymer loading. Briefly, this custom mechatronic device contains a DC brushless motor, wherein microfluidic devices can be attached via hex screws and spun to centrifugally move fluid. The spin aspect of the system is controlled by Parallax software.

#### 2.2.2 Single‐color electrophoresis system

A custom‐built single‐laser, single‐color fluorescence detection system was designed for electrophoretic separation and laser‐induced fluorescence (LIF) detection (Figure S2). The microdevice is placed on a metal stage that can be heated for electrophoresis and adjusted in three dimensions to focus the incident laser through a hole in the stage into the middle of the separation channel of the microfluidic device. Voltage‐driven electrophoresis was achieved by placing platinum electrodes connected to a high‐voltage power supply in the 3D printed reservoirs on the microfluidic chip seated on the metal stage. The custom‐built voltage supply system is controlled by LabVIEW. The fluorescence detection system consisted of a 488‐nm solid‐state laser for excitation, which was directed to a mirror, through a 525‐nm short pass dichroic mirror, and into the end of a 40× LD Acroplan, 0.6 NA objective (Zeiss, Thornwood, NY, USA) where it focused into the microdevice separation channel. The emission beam was passed back through the objective, reflected off the dichroic mirror, and passed through a 505‐nm long pass filter toward a photomultiplier tube detector (Hamamatsu, Tokyo, Japan). Data collection and electrophoresis conditions were controlled by a custom‐made LabVIEW program.

### 2.3 Evaluation of autofluorescence

The custom‐built fluorescence detection system described previously was used to evaluate inherent fluorescence in raw polymeric material for microfluidic chip fabrication. Sheets of raw materials were placed over the hole on the metal stage, and the incident laser beam was focused on the sheet via the objective. Once the excitation beam was focused, the sheet was removed. Data collection began for 10 s to collect ambient noise, and then the sheet of polymeric material was placed onto the stage to measure any inherent fluorescence of the material.

### 2.4 Sample extraction and amplification

Samples were prepared following a previously described protocol [12]. Briefly, DNA from a buccal swab containing saliva was enzymatically extracted using MicroGEM reagents (MicroGEM International, PLC, VA, USA). Extracts underwent PCR amplification in‐tube using a custom 10‐plex kit that amplified 9 core CODIS loci. The resulting amplicons were under 250‐base pairs in length and consisted of markers composed of FAM, JOE, or ROX (ET‐CRX). Sample was amplified conventionally following manufacturer‐recommended thermal cycling settings for the PowerPlex Fusion System.

### 2.5 Microchip preparation

Following fabrication, the microchip for electrophoresis was pretreated with 0.1‐M NaOH (Sigma‐Aldrich, MO, USA) for 10 min prior to loading polymer. The NaOH was removed via centrifugation on the DIPS system (180 s, 1600 RPM). A hydrophobically modified poly(acrylamide) polymer (MicroGEM International, PLC, VA, USA) consisting of 4% (w/v) in 3× Tris–borate–EDTA (TBE, 49 mM Tris, 147 mM borate, 1 mM EDTA, Sigma‐Aldrich, 7 M urea, and ultrapure water) was pipetted into the BW reservoir and centrifugally loaded (180 s, 1600 RPM) through the separation architecture. The devices were heated at 50°C for 10 min and then spun for 60 s at 1600 RPM to remove any bubbles. Once all reservoirs were filled with polymer sufficiently for voltage application, the sample was pipetted into the sample reservoir. A stock solution of fluorescein (0.01 M FAM; Sigma‐Aldrich, MO, USA) was diluted in 1× TBE and used as a reference dye in place of sample when evaluating the shape of the injection plug via a fluorescence microscope (ZEISS Axioscope, Germany).

### 2.6 Microchip electrophoresis

Following amplification, sample (5 µl) and size standards (5 µl) were mixed with Hi–Di formamide (10 µl). The mixed sample was heated at 95°C for 2 min and then immediately placed in ice for 2 min to maintain single‐stranded fragments. Sample was then pipetted into the 3D printed sample reservoir of the polymer‐filled chip. The electrophoresis chip was then placed on the heated stage (47°C) of the single‐color electrophoresis system with the incident laser beam focused 4 or 6 cm from the cross‐T intersection, depending on the chip being used. Platinum electrodes were placed in each of the S, SW, B, and BW wells, and injection voltages of −100 and +100 V were applied to the S and SW reservoirs, respectively, for 90 s. After injection, −200 V was applied to the B reservoir, +800 V was applied to the BW reservoir, and both the S and the SW electrodes were grounded. This provided a pull‐back mechanism to limit plug size and sample leakage.

## 3 RESULTS AND DISCUSSION

### 3.1 Microdevice fabrication

To fabricate the microfluidic discs for electrophoretic separations, the PCL technique previously reported by our lab was employed [7]. Briefly, the PCL method utilizes a CO_2_ laser cutter to define microfluidic architecture in sheets of a printable substrate. A laser printer was then used to deposit printer toner to act both as an adhesive agent for bonding and create the microfluidic architecture. This technique allows microdevices to be rapidly and inexpensively prototyped with a design‐to‐device time of ∼40 min using device materials that cost less than 1.00 USD [5]. The original PCL method utilized polyethylene terephthalate (PeT), commonly known as “overhead transparencies,” for all layers of the microfluidic device, as this substrate was inexpensive, yet effective for a variety of applications [5, 8, 13, 14].

**FIGURE 1:**
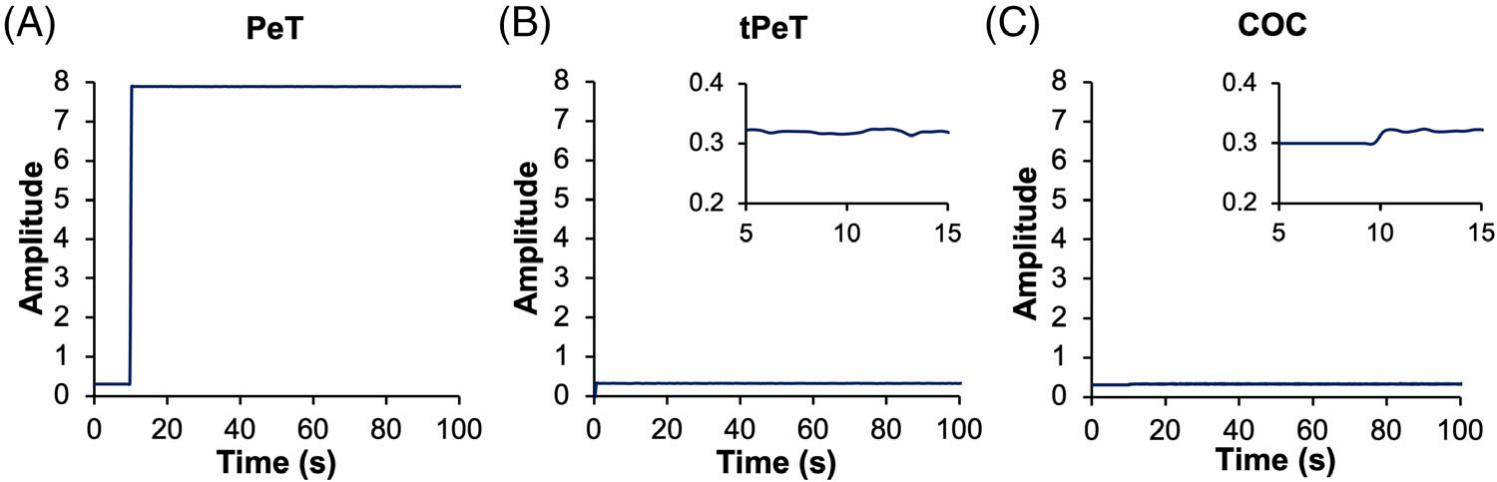
Inherent fluorescent emissions of candidate substrates for microfluidic device fabrication when excited by a 488‐nm sapphire laser: (A) signal from a sheet of polyethylene terephthalate (PeT), (B) toner‐coated PeT (tPeT), and (C) cyclic olefin copolymer (COC). Insets in (B) and (C) illustrate any change in the signal at 10 s when the substrate was placed on the custom electrophoresis system with a fluorescence detector

When creating a device for electrophoresis with fluorescence detection, the optical properties of the substrate used are of obvious import; any inherent fluorescence, or autofluorescence, from the substrate will be captured by the detector and, if intense, potentially overpower the fluorescence signal from analytes of interest. For this reason, we tested the autofluorescence of PeT relative to a series of other substrate candidates for electrophoresis by exposing a thin sheet to a 488‐nm laser in a custom single‐color electrophoresis system containing hardware for LIF detection (Figure 1). The detector collected ambient noise (no substrate) for 10 s prior to exposing the desired material to the laser; after the initial placement of the substrate, fluorescence signal did not significantly change (i.e., no autofluorescence was observed). PeT exhibited intense inherent fluorescence, extensive enough to reach and maintain the maximum output at the detector, making this material a poor candidate for an electrophoresis device. When coated with toner, PeT does not exhibit any autofluorescence beyond ambient noise (Figure 1B); however, although tPeT is a good candidate for an internal layer within a microdevice, it cannot be an outer layer because the toner printing is reversible and, thus, would eventually expose PeT. Given this, we explored an alternative polymeric material, COC, which had previously been demonstrated to be compatible with the PCL method and could act as capping layers, or the top and bottom, of the microdevice. As expected from the literature [15, 16], excitation of COC with 488‐nm light resulted minimal fluorescence above ambient noise (Figure 1C); thus, COC was chosen for future device fabrication for both the top and bottom layers. This is consistent with the widespread use of COC where optical detection is desired and previous reports using injection‐molded COC for DNA separations, which cite optical clarity as one of the advantages [16, 17]. It is worthy of note that bonding COC is not trivial [18, 19]; hence, we explored methods for the effective bonding of COC to the top and bottom of the tPeT layer. As illustrated in Figure S1, the tPeT middle layer will have laser ablated microfluidic architecture and be sealed by a top and bottom layer of COC, wherein necessary vents, alignment holes, and ports for fluidic access will be laser ablated.

### 3.2 Device bonding

As a capping layer, COC possesses excellent optical properties for fluorescence detection and a surface that is ideal for polymer‐based electrophoresis; however, methods for bonding between COC and tPeT needed to be explored and optimized. Following the original PCL method, a microfluidic disc containing four identical electrophoresis channels was fabricated by stacking tPeT between two COC layers and laminating at 200°C to activate the toner as an adhesive. Following exposure to even slight hydraulic pressure, it was clear upon inspection that all three layers had easily delaminated (Figure S3A). It was clear that toner failed to form an efficient bond between the PeT and COC layers. This is likely explained by the hydrophobic nature of the COC surface (contact angle ∼80°), but this can be modified substantially through plasma oxidation, decreasing the contact angle <5° after 10 min of exposure [20]. Based on this, all three layers were plasma oxidized for 10 min prior to alignment and lamination to modify the surfaces (Figure S3B). The uncoated COC sheets showed a dramatic improvement in bonding to the tPeT layer; when tested, the layers were no longer delaminated without damaging the device. Plasma oxidation of layers prior to lamination was carried out for all further fabrication.

### 3.3 Architectural design of separation chip

**FIGURE 2:**
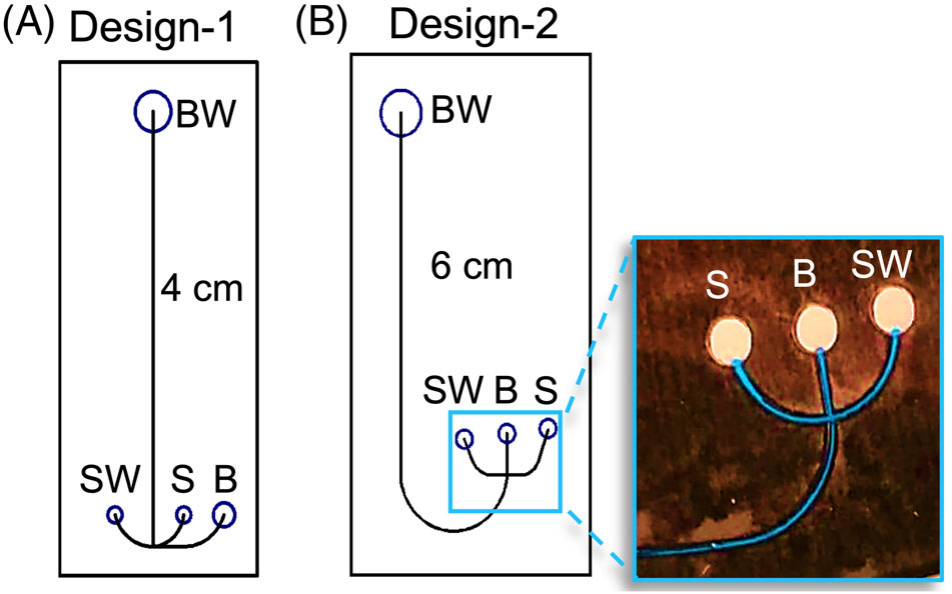
Prototype electrophoresis microchip architectures: (A) microchip with an “anchor”‐like cross‐T and a 4‐cm effective separation length (*L*_eff_); (B) chip containing a 6‐cm *L*_eff_ and a traditional cross‐T design. Inset shows the cross‐T filled with polymer mixed with a blue dye for imaging purposes

After optimizing the bonding procedure for the electrophoretic microdevice, microfluidic architectures amenable to centrifugal reagent loading were explored to supplant the need for pumps or external pressure‐generating hardware. With the desire to centrifugally load the electrophoretic microchips with polymer, they were fabricated with designs differing in the effective separation length (*L*_eff_) and cross‐T architecture (Figure 2). Different width and depth dimensions were not explored, as these were defined by the resolution of the laser cutting (∼100 µm) and the thickness of the tPeT layer (∼124 µm), respectively. Design‐1 consisted of a straight separation channel with a 4‐cm *L*_eff_ and a unique “anchor‐like” cross‐T design (Figure 2A), whereas Design‐2 had a 6‐cm *L*_eff_, but with a 3.75‐cm straight channel prior to curving to a more traditional cross‐T (Figure 2B). Centrifugal reagent loading capability was tested with a hydrophobically modified polyacrylamide polymer (4% w/v) that had previously been shown effective for DNA separations [21]; this contained blue dye for imaging purposes and was pipetted into the BW reservoir. The microfluidic chip was then placed on a DIPS system with the BW reservoir closest to the center of rotation. Spin speeds up to 3000 RPM were tested with varying spin times up to 180 s. It was determined that 1600 RPM for 180 s was sufficient for effective centrifugal polymer loading, as indicated by the complete filling of all channels and the cross‐T to the base of the sample (S), SW, and buffer (B) reservoirs, as shown in the in Figure 2B inset.

### 3.4 Sample plug evaluation

**FIGURE 3:**
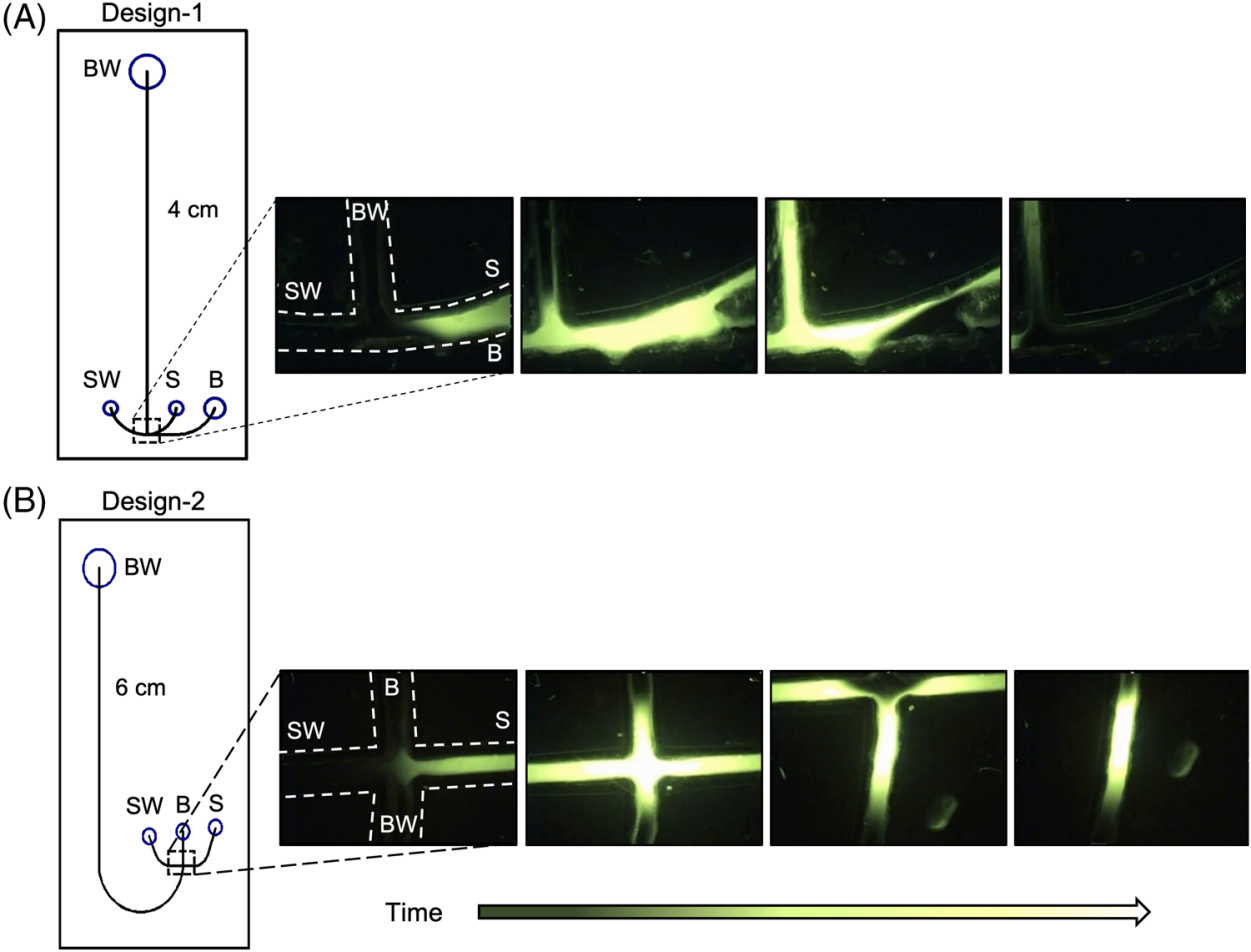
Evaluation of the injection plug shape using fluorescein: (A) a series of images showing the injection of fluorescein in the “anchor”‐like cross‐T of the 4‐cm effective separation length (*L*_eff_) chip; (B) the 6‐cm *L*_eff_ chip with fluorescein injected across the traditional cross‐T. For both conditions, −/+100 V were applied to the S and sample waste (SW) reservoirs for a 90‐s injection, then −200 V was applied to the B reservoir and +800 V was applied to the buffer waste (BW) reservoir, whereas both the S and SW reservoirs were ground

Prior to performing electrophoretic separations on the microfluidic chips, the injected sample plug was evaluated because the sample plug shape ultimately impacts resolution (Figure 3). For these experiments, the hydrophobically modified poly(acrylamide) polymer was injected into the BW, centrifugally driven through the channels and arms of the cross‐T, and fluorescein was added to the sample reservoir. Electrophoresis occurred using voltage power supply in our custom in‐house built single‐color system, and imaging was conducted with a fluorescent microscope. Figure 3A depicts the “anchor‐like” design injecting an undesirably large and uneven sample plug shape into the separation channel. Comparatively, the injected sample plug seen with Design‐2 appears similar to the shape of traditional sample plugs (Figure 3B). As both designs have a short separation length (relative to traditional electrophoresis and other microchip electrophoresis designs), and large sample plugs have been shown to have a negative effect on the resolution in a given separation [22], electrophoresis was carried out on the chip containing the traditional cross‐T design and the 6‐cm *L*_eff_.

### 3.5 Evaluating separation resolution

After optimizing the fabrication method, microfluidic architecture, reagent loading, and ensuring the sample plug was similar to that seen in traditional electrophoresis, a 400‐base DNA sizing ladder was separated on the 6‐cm *L*_eff_ microchip with a traditional cross‐T, and the resulting electropherogram was used to calculate separation resolution. Electrophoretic resolution (*R*_s_), a key metric used to assess and compare separation performance, was calculated according to the following equation:

(1) $$R_{S}=2\;\frac{\Delta t_{m}}{W_{b1}+W_{b2}}$$

where *R*_s_ is defined by the ratio of the difference in migration time between two adjacent peaks (Δ*t*_m_) and the average base width of the peaks (*W*_b1_ + *W*_b2_)/2. The base‐pair resolution between peaks at full width half maximum (*R*_bp_) normalizes the separation in terms of base pairs to determine the smallest difference in fragment length that can be resolved by the system. The resolution per base pair is defined by the size difference between two fragments (Δ*N*) and the width of the peak (*W*_h_), as shown in the following equation [23]:

(2) $$R_{\mathrm{bp}}=\frac{\Delta t_{m}}{\Delta NW_{h}}$$

**FIGURE 4:**
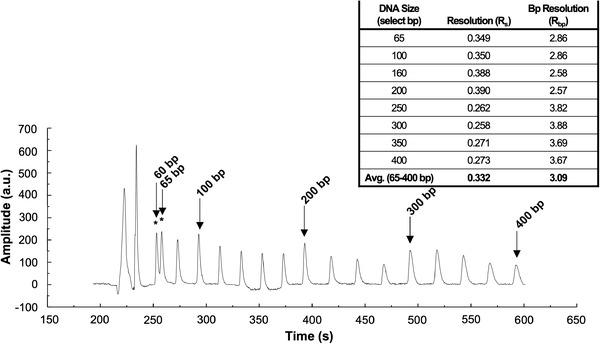
Electropherogram depicting separation of a 400‐base DNA size ladder showing differences in size as small as 5 bases and as large as 25 bases. Injection voltages of −100 and +100 V were applied to the S and sample waste (SW) reservoirs, respectively, for 90 s. Following injection, −200 V was applied to the B reservoir and +800 V was applied to the buffer waste (BW) reservoir, whereas both the S and the SW electrodes were ground. The inset table displays resolution (*R*_s_) and base pair (bp) resolution (*R*_bp_) calculated using PeakFit analysis software

An exemplary profile is shown in Figure 4, where peak analytes differing in molecular size by as few as 5 bases (peaks with *) were observed. PeakFit analysis software determined the calculated *R*_s_ and *R*_bp_ was ∼3 bases with DNA fragments <250 bases, whereas fragments >250 bp showed ∼4 base resolution (Figure 4 inset). This represents a ∼14–18‐fold improvement in resolution over previously reported DNA separations on similar substrates consisting of a 200‐µm wide and 12‐µm high separation channel with a 40‐cm *L*_eff_ [11]. It is worth noting that although voltages of 200 V/cm were applied during this experiment, higher voltages (up to 400 V/cm) have been applied to these single‐use/disposable chips in previous studies; however, the application of voltage above this causes Joule Heating.

### 3.6 On‐chip electrophoresis

Finally, to demonstrate the accuracy and precision for the separation and sizing of DNA fragments on this PCL device, DNA amplicons were electrophoresed. The FAM‐labeled products resulting from off‐chip PCR amplification of STR sequences included the D3S1358 and D13S317 loci in a human buccal swab.

**FIGURE 5:**
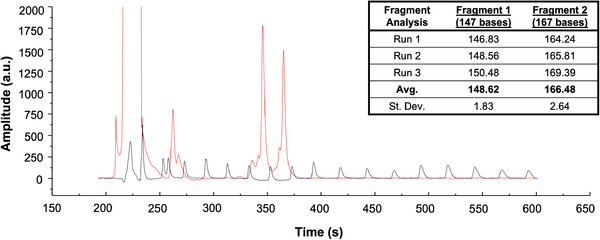
Electropherogram from on‐chip electrophoresis completed in 10 min. Injection voltages of +/−100 V were applied to the S and sample waste (SW) reservoirs for 90 s, then −200 V was applied to the B and +800 V was applied to the buffer waste (BW) reservoir, whereas the S and SW reservoirs ground. PCR‐amplified DNA fragments (red) overlaid with a 400‐base DNA size ladder (black) (*n* = 3). The amplified fragments had expected sizes of 147 and 167 bases, respectively, and the average calculated size of each fragment was 148.62 ± 2 and 166.48 ± 3 bases (inset)

Hydrophobically modified poly(acrylamide) polymer was centrifugally loaded into the separation architecture and reservoirs, and the PCR sample was pipetted into the sample reservoir prior to being electrokinetically injected through the cross‐T. The custom‐built single‐color electrophoresis system, containing a hardware for LIF detection (ex. 488 nm; em. 505–545 nm), was used with the Design‐2 microfluidic chip (Figure S2). The microdevice was placed on a 47°C heated stage to yield exemplary electropherograms, one of which is shown in Figure 5. Here, the separation of a sample containing the 400‐base DNA ladder, shown in Figure 4, is spiked with D3S1358 and D13S317 loci PCR amplicons. First, note the separation, including a 90‐s electrokinetic injection, is completed in less than 10 min (Figure 5). Second, the reproducibility from the separation of the ladder standards is clearly illustrated in Figure S4, which depicts well‐aligned amplicon peaks obtained in individual microchip electrophoresis replicates. Third, using the sizing ladder to calculate amplicon base pair length for three consecutive runs, the average amplicon size and corresponding standard deviation were determined to be 148.62 ± 2 (D3S1358) and 166.48 ± 3 (D13S317) bases (Figure 5 inset). This correlates well with the known size for the D3S1358 and D13S317 fragments at 147 and 167 bases, respectively. These results illustrate the capability of COC‐tPeT hybrid electrophoretic devices fabricated by PCL, where the cost of materials is <1.00 USD and the time to fabricate is <30 min. Given the accuracy and precision of these results with 3–4 base resolution in sub‐10‐min separation, this positions laser‐cut microchannels as effective for DNA separations where single‐base resolution is not required.

## 4 CONCLUDING REMARKS

Here we demonstrate that the limitations reported in prior literature for DNA fragment analysis on PeT microdevices can, in fact, be overcome. After examining autofluorescence in different materials, and selecting a combination of materials to enable successful fluorescence detection of polynucleic acids, a microchip consisting of laser‐cut COC surrounding one tPeT layer with fluidic architecture was created. Bonding was enhanced through plasma oxidation prior to lamination of the device, and separation architectural designs were evaluated based on amenability to centrifugal reagent loading. It was determined that chip Design‐2, containing a 6‐cm curved separation channel with a semi‐traditional cross‐T, was the better design due to the ability to rotationally load polymer in a reasonable amount of time. Further, the shape of the injection plug was similar to standard injection plugs. Finally, we demonstrated successful separation of a DNA sizing standard with <3 base pair resolution for <250 base pairs and <4 base pair resolution for >250 bases. Not only is this a ∼14–18‐fold improvement in resolution over previously reported DNA separations on similar microdevices, but the injection and separation were complete in under 10 min. The accurate and precise sizing of two STR makers was illustrated by separating DNA fragments of 147 and 167 bases (in less than 10 min) and sizing them as 148.6 ± 2 and 166.48 ± 3 bases, respectively. This report shows that PCL devices, which cost <1.00 USD and require <30 min to fabricate, are able to perform precise polynucleic acid separation in an inexpensive, rapidly prototyped microfluidic electrophoresis chip.

## CONFLICT OF INTEREST

The authors have declared no conflict of interest.

## Supporting information

Supporting Information

Supporting Information

Supporting Information

Supporting Information

Supporting Information

## Data Availability

The data that support the findings of this study are available from the corresponding author upon reasonable request.
