# Supplementary material for: Rapid, inexpensive fabrication of electrophoretic microdevices for fluorescence detection
Source: Electrophoresis. 2022 Jul 8;43(16-17):1746–54. doi: 10.1002/elps.202200090 (PMC9544361; doi:10.1002/elps.202200090)
Supplement: Supplementary file 1 — Supporting Information [file ELPS-43-1746-s004.docx]

**Supplementary Material**

**Rapid, Inexpensive Fabrication of Electrophoretic Microdevices for Fluorescence Detection**

Daniel A. Nelson^1^, Renna Nouwairi^1^, Brandon L. Thompson^1^, An-Chi Tsuei^1^, Christopher Birch^1^, Jacquelyn A. Duvall^1^, Delphine Le Roux^1^, Jingyi Li^1^, Brian E. Root^1^, James P. Landers^1,2,3^

^1^ Department of Chemistry, University of Virginia, Charlottesville, VA, USA.

^2^ Department of Mechanical Egineering, University of Virginia, Charlottesville, VA, USA.

^3^ Department of Pathology, University of Virginia, Charlottesville, VA, USA.


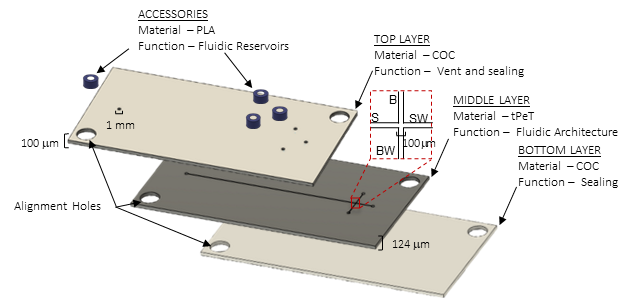


**Figure S1**: **Exploded view of the three-layer microfluidic electrophoresis chip with dimensions.** Microchip consisting of toner-coated polyethylene terephthalate (tPeT) sandwiched between two layers of cyclic olefin copolymer (COC). Accessory pieces include 3D printed reservoirs, made from polylactic acid (PLA) and attached to the chip via epoxy. All three layers contain alignment holes for accurate, consistent layer stacking.


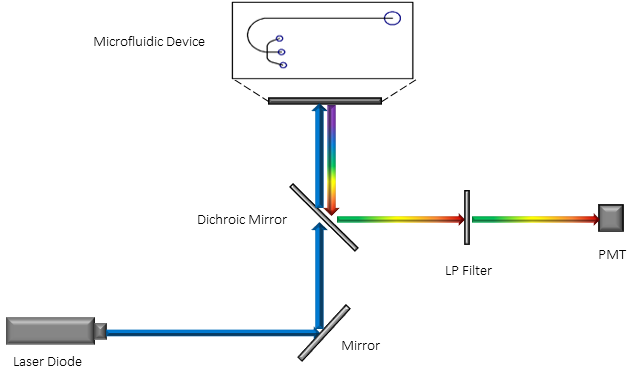


**Figure S2. The set-up of the single-color electrophoresis system**. A 488 nm solid-state sapphire laser is reflected off a mirror, through a 525 nm dichroic mirror, and onto the microfluidic device. Light is reflected from the device back to the dichroic mirror then passed through a 505 nm long pass (LP) filter and to a photomultiplier tube (PMT) for signal amplification.


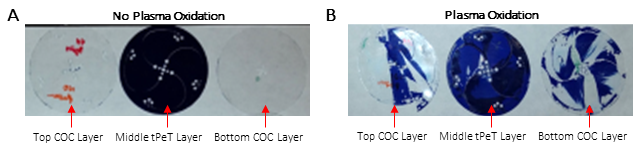


**Figure S3. Three-layer disc pulled apart following fabrication via toner coating, laser ablation, and lamination.** A) Disc bonded only by lamination. B) Disc layers plasma oxidized for 10 min prior to lamination.


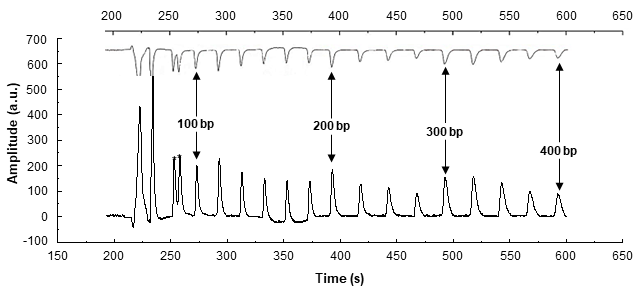


**Figure S4. Comparison of electropherograms from different runs illustrating the reproducibility of separations.** Separation of a 400-base DNA size ladder completed across different runs but overlayed to illustrate reproducible fragment sizing. The graph on the top was collected when separating a ladder spiked with sample; the lower graph is from separation of only a ladder.
