# Supplementary figures and images for: Rapid, inexpensive fabrication of electrophoretic microdevices for fluorescence detection
Source: Electrophoresis. 2022 Jul 8;43(16-17):1746–54. doi: 10.1002/elps.202200090 (PMC9544361; doi:10.1002/elps.202200090)

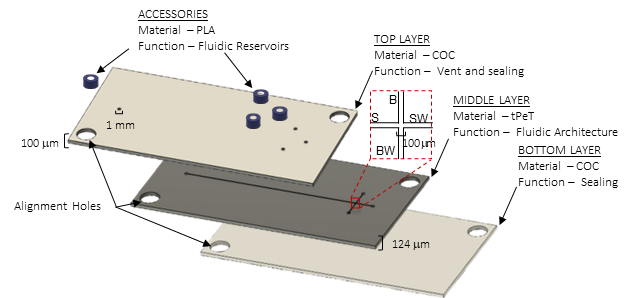

Supplement: Supplementary file 2 — Supporting Information [file ELPS-43-1746-s005.tif]

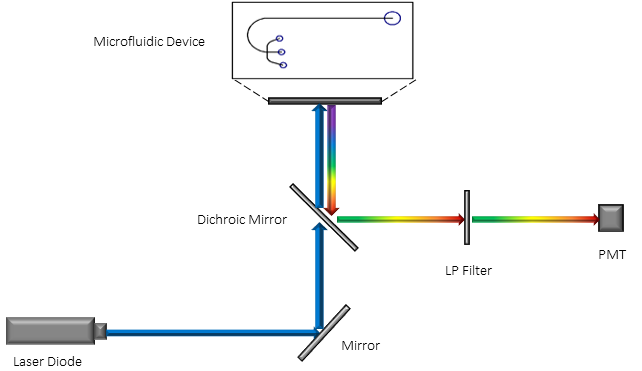

Supplement: Supplementary file 3 — Supporting Information [file ELPS-43-1746-s001.tif]

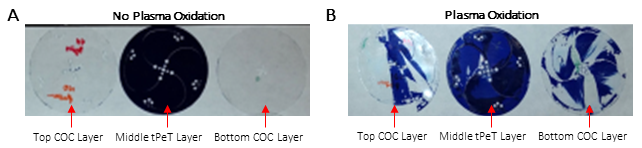

Supplement: Supplementary file 4 — Supporting Information [file ELPS-43-1746-s002.tif]

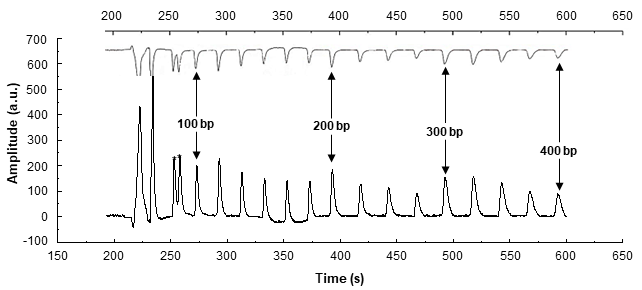

Supplement: Supplementary file 5 — Supporting Information [file ELPS-43-1746-s003.tif]
